# Use of Blue Light in the Management of Chronic Venous Ulcer in Asian Patients: A Case Series

**DOI:** 10.7759/cureus.17703

**Published:** 2021-09-04

**Authors:** Vanessa B Khoo, Shereen Soon, Charyl J Yap, Siew Ping Chng, Tjun Y Tang

**Affiliations:** 1 Department of Vascular Surgery, Singapore General Hospital, Singapore, SGP

**Keywords:** photobiomodulation, wound healing, venous ulcer, blue light, laser therapy

## Abstract

Chronic venous ulcers, often complicated by late diagnosis and persistent infections, present major clinical and financial challenges. Recently, photobiomodulation therapy (PBMT) has been shown to be effective in overcoming physiological impairments such as hemostasis and inflammation, accelerating the wound healing process. This case series summarises our experience in the treatment of two Asian patients with lower-extremity chronic venous ulcers using PBMT with blue light. Case 1 was a 71-year-old male with a history of hypertension, chronic venous insufficiency and previous deep vein thrombosis. Prior to blue light therapy, the average duration of treatment until wound closure with compression dressings used to be 10-12 weeks. Complete wound closure with the blue light therapy was eight weeks, with a total reduction of 67% of wound size by week 4. Case 2 was a 77-year-old male with a background of hypertension and ischaemic heart disease. Prior to blue light therapy, the patient had also underwent iliac venoplasty and stenting for his recurring bilateral malleolus venous ulcers. By week 4, the right malleolus wound had healed, while the left malleolus wound had a size reduction of 38%. Complete closure of both the wounds was noted at week 6. Blue light was administered to the wounds of both patients for 120 seconds per session, as an adjunct to compression therapy. Both patients reported no additional wound pain during the administration of blue light therapy, with an overall reduction of wound pain at three weeks. The cases demonstrated that PBMT with blue light was well-tolerated, safe, and efficacious in improving wound healing with an adjunct to standard treatment of chronic venous ulcers.

## Introduction

Chronic venous ulcers present major clinical and financial challenges. The global prevalence of venous ulcers is estimated to be 0.6%-2% [1]. In the UK, treatment of venous ulcers has been shown to cost approximately 4% of the country’s healthcare spendings per year [2]. In developed countries, these figures are also projected to increase with the rising rates of comorbidities such as diabetes and hypertension [3]. Unlike acute wounds, patients with chronic ulcers tend to have impairment in one or more physiological processes of healing, such as hemostasis and inflammation [4]. These impairments result in persistent wound infections, the lack of wound closure (over six weeks) or wound recurrence in patients with newly healed wounds.

Therapeutic challenges in treating chronic venous ulcers include early diagnosis and the use of the appropriate therapeutic device for wound healing. Photobiomodulation therapy (PBMT) involves the use of photons from specific frequencies of the visible light spectrum to stimulate the wound healing process [5]. In recent years, there has been an increasing demonstration of the benefits of PBMT for wound healing in the literature such as promoting vasodilation, angiogenesis, stimulation of cellular respiration, fibroblast activity, and increased connective tissue repair [6]. The use of blue light (wavelength 400-450 nm) in particular has also been shown to have anti-inflammatory properties, reducing bacterial load and the release of proinflammatory cytokines.

In this case series, we report observations made from the treatment of blue light (EmoLED srl, Florence, Italy) in two patients diagnosed with recurrent venous ulcers of the lower limbs, in addition to the standard treatment of care at a tertiary vascular center in Singapore.

## Case presentation

PBMT with blue light (EmoLED device)

**Figure 1:**
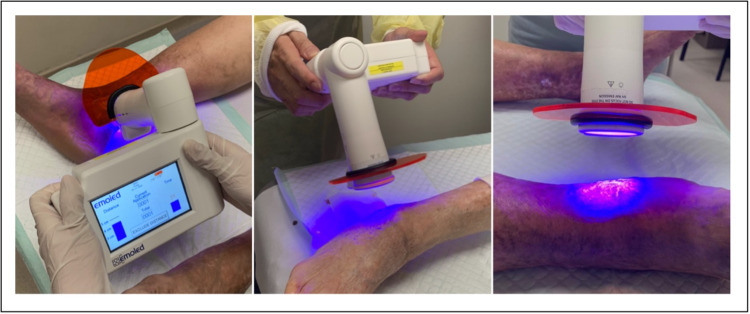
Administration of blue light therapy using EmoLED. Treatment with EmoLED was administered to the wounds once a week for 120 seconds in an outpatient setting, complementary to the standard of care adopted prior to blue light therapy.

The medical device EmoLED uses LED sources emitting an area of 20cm² of blue light (wavelength 400-430 nm), with a T power density of 120 mW/cm² and a fluence of 7.2 J/cm²​​​​​​​ at 4cm from the light source [7]. Treatment with EmoLED was administered to the wounds once a week for 120 seconds in an outpatient setting, complementary to the standard of care adopted prior to PMBT, including compression therapy and wound dressings (Figure 1).

Case 1

**Figure 2:**
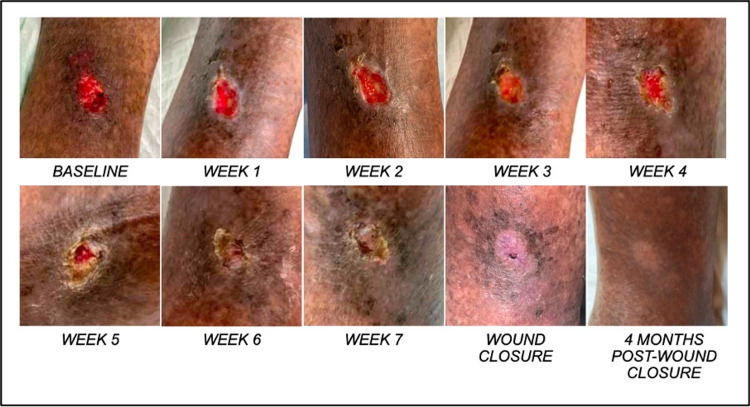
Patient 1 wound healing progression with weekly blue light therapy on left shin wound.

A 71-year-old Chinese male with recurrent bilateral lower limb venous ulcers since 2018 and a background of chronic venous insufficiency. He has a past medical history of hypertension, atrial fibrillation, cervical spondylosis and left-sided deep vein thrombosis. Prior to blue light therapy, the patient underwent bilateral great saphenous vein ablation with cyanoacrylate glue in March 2019 and left Iliac vein venoplasty and stenting in April 2020 for his recurrent ulcers. The recurrent ulcers were also typically treated with weekly four-layer compression dressing (average duration of treatment until wound closure, 10-12 weeks for each recurrence). He developed a new ulcer on his left lateral shin eight months after, requiring further surgical debridement. Post-debridement, he was placed on weekly two-layer compression dressing. Prior to the administration of blue light therapy, his ulcer had already recurred for four weeks. Blue light therapy was administered seven times across 8 weeks, as an adjunct to the patient’s weekly two-layer compression dressing. Each dose of blue light therapy was administered at a height of 4 cm from the wound for 120 seconds, with an irradiated area of 20 cm²​​​​​​​. The baseline measurement of the wound size was 3cm² (1.5 cm x 2 cm, Figure 2). By week 5, there was a total reduction of 67% of wound size (week 4; 1 cm², 1 cm x 1 cm). The patient also reported a reduction in wound-pain experienced while performing activities of daily living. Complete wound closure was observed at week 8. No wound recurrence was reported at up to four-month follow-up post-wound closure.

Case 2

**Figure 3:**
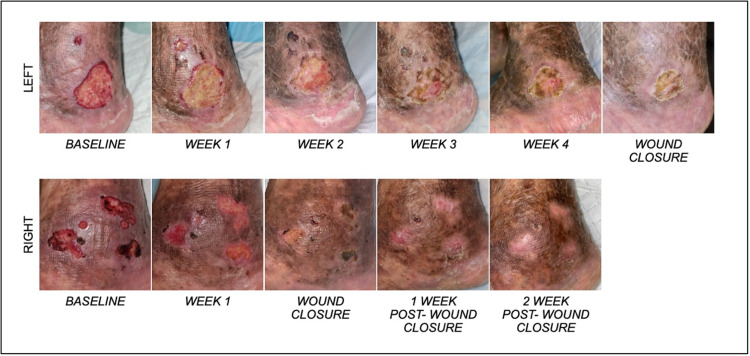
Patient 2 wound healing progression with weekly blue light therapy on left lateral malleolus (top), and right medial malleolus (bottom) wound.

A 77-year-old Chinese male with multiple non-healing venous ulcers on his lower extremities and a background of chronic venous insufficiency. He has a past medical history of hypertension with cardiac right bundle branch block, and vitamin B12 deficiency. The onset of the non-healing venous ulcers dated back to early 2017. Prior to the blue light therapy, the patient was on a four-layer compression dressing and underwent iliac venoplasty and stenting in 2019. The recurrent ulcers remained healed for a year before recurring in 2021. Prior to blue light therapy, his ulcers had already recurred for three weeks. Adjunct with the patient’s weekly dressing change, blue light therapy was administered for 120 seconds on both the left lateral malleolus wound and the right medial malleolus wound, until wound closure. Each dose of blue light therapy was administered at a height of 4cm from the wound, with an irradiated area of 20 cm²​​​​​​​. Regions of the ulcer that fell outside of the irradiated area, were administered to in subsequent rounds. The baseline wound size of the right medial malleolus and left lateral malleolus were 13.5 cm² and 10.5 cm², respectively (Figure 3). By week 3, the right medial malleolus wound had healed, while the left lateral malleolus had a size reduction of 37.8% (week 4; 6.25 cm²). Complete closure of the left malleolus wound was noted 6 weeks after the use of blue light therapy.

## Discussion

PBMT

The use of PBMT for wound healing dates back to the 1900s with the use of laser, before the emergence of light emitting diodes (LEDs). PBMT have shown beneficial effects on both healing stimulation and ulcer cleansing. It provides benefits in tissue repair with a series of effects such as increased capillary and vascular circulation, stimulation of cellular respiration, fibroblast activity with increased collagen deposition and connective tissue repair [6]. The different light-tissue interactions amongst light of various wavelengths determines their appropriateness and effectiveness in treating wounds of differing etiology. Red and blue PBM wavelengths (620-750 nm and 435-500 nm, respectively) are more commonly explored for superficial tissues (lower penetrance, higher absorption), while near-infrared (750-950 nm) wavelengths are usually preferred to treat more deep-seated tissues (high penetrance, lower absorption) [8].

In the literature, however, data on the use of PBMT on chronic wounds is scarce. Optimal and standardized clinical protocols on the administration of the various types of PBMT on differing wounds are also lacking amongst clinicians. A review article revealed that there had only been 11 articles pertaining to the use of PBMT on chronic would healing between 2013 and 2018 [5]. Despite limitations in sample sizes and heterogeneity, these studies have shown promising benefits of wound healing such as the reduction of wound size, wound pain, inflammation, re-epithelization and improved healing rates in burn wounds, diabetic foot, venous and pressure ulcers.

PBMT with blue light (EmoLED device)

Including those presented in the cases, we have had seven patients with non-healing/recurrent venous ulcers (> 4 weeks) on PBMT with blue light therapy using the EmoLED device (not FDA regulated). All patients were reported to have high pain-scores and exudative wounds. PBMT was administered weekly in an outpatient setting in conjunction with their standard treatment such as compression therapy. Patients have reported to be very satisfied with the treatment of blue light therapy, having seen marked improvements in wound healing rates in comparison to the wound healing durations from prior recurring wounds. Our experience with EmoLED blue light therapy and its impact on wound healing rates has also been compared to the case studies of the Caucasian cohorts where PBMT with blue light was administered as an adjuvant to compression therapy, to a pool of patients with recurrent venous ulcers and similar comorbidities of chronic venous insufficiency [6,7]. 

Despite the small patient pool, the results from this case series and cases in the literature are encouraging, showing faster healing trajectories with blue light therapy administered adjuvant to compression therapy in comparison to compression therapy alone. There is now a need for randomised controlled trials to properly assess the efficacies that we have observed in the use of PBMT with blue light adjuvant to compression therapy versus standard of care compression therapy alone for venous ulcers.

## Conclusions

The use of PBMT with blue light in conjunction with standard treatment in the Asian population with chronic venous ulcers is safe, well-tolerated, cost-efficient, and efficacious in promoting wound healing especially in patients with chronic lower limb venous ulcers and a history of cardiovascular disease. Results from the current literature and our small pool of patients are encouraging, and have prompted us to participate in a protocol of a prospective study on the efficacy of blue light in lower limb lesion management for Asian patients with diabetes (BLOOM study).

## References

[1] Santoso ID, Nilasari H, Yusharyahya SN (2017). Venous ulcer. J Gen-Procedural Dermatol Venereol Indonesia.

[2] Guest JF, Ayoub N, McIlwraith T (2017). Health economic burden that different wound types impose on the UK's National Health Service. Int Wound J.

[3] Goh OQ, Ganesan G, Graves N, Ng YZ, Harding K, Tan KB (2020). Incidence of chronic wounds in Singapore, a multiethnic Asian country, between 2000 and 2017: a retrospective cohort study using a nationwide claims database. BMJ Open.

[4] Loh C, Tan QY, Eng DL, Walsh SR, Chong TT, Tang TY (2021). Granulox-The Use of Topical Hemoglobin to Aid Wound Healing: A Literature Review and Case Series From Singapore. Int J Low Extrem Wounds.

[5] Mosca RC, Ong AA, Albasha O, Bass K, Arany P (2019). Photobiomodulation therapy for wound care: a potent, noninvasive, photoceutical approach. Adv Skin Wound Care.

[6] Mosti G, Gasperini S (2018). Observations made on three patients suffering from ulcers of the lower limbs treated with Blue Light. Chronic Wound Care Management and Research.

[7] Marchelli M, Perniciaro G, Granara D (2019). Photobiomodulation with Blue Light in non-healing wounds: case series evaluation. Wounds Int.

[8] Anders JJ, Lanzafame RJ, Arany PR (2015). Low-level light/laser therapy versus photobiomodulation therapy. Photomed Laser Surg.

